# Psychological Shift in Partners of People with Multiple Sclerosis Who Undertake Lifestyle Modification: An Interpretive Phenomenological Study

**DOI:** 10.3389/fpsyg.2018.00015

**Published:** 2018-01-31

**Authors:** Sandra L. Neate, Keryn L. Taylor, George A. Jelinek, Alysha M. De Livera, Chelsea R. Brown, Tracey J. Weiland

**Affiliations:** Neuroepidemiolgy Unit, Melbourne School of Population and Global Health, University of Melbourne, Melbourne, VIC, Australia

**Keywords:** multiple sclerosis, qualitative research, partners, lifestyle intervention, adaptation, empowerment, hope, taking control

## Abstract

**Introduction:** Being in an intimate relationship with a person with multiple sclerosis (MS) may have a substantial impact on the partner's quality of life. Existing research has largely focused on negative impacts of MS for both people with MS (PwMS) and their partners and has sampled the population of partners of PwMS who have primarily adopted standard medical management only. Modifiable lifestyle factors have become increasingly recognized in the management of MS symptoms and disease progression. For partners of PwMS who have undertaken lifestyle modification as an additional strategy to minimize disease progression, the impacts, both positive and negative remain unexplored. This research is unique as it focuses on partners of PwMS who have attempted to adopt major lifestyle interventions outside of the prevailing paradigm of MS management.

**Aim:** To explore and interpret the lived experiences of partners of PwMS who have adopted lifestyle modification, to understand partners' attitudes to and experiences of the effect of MS and lifestyle modification on their life, relationship and view of the future.

**Method:** Design: a qualitative, interpretive, phenomenological study using semi-structured interviews. Participants: English-speaking; aged 18 years or more; in a spousal relationship for 12 months or more with a person with MS who had attended a residential lifestyle educational intervention and undertaken lifestyle modification. Analysis: Interviews were recorded, transcribed verbatim and thematically analyzed using NVivo™ software.

**Results:** Twenty-one partners were interviewed. This paper reports one of the study's themes, the psychological shift experienced by partners of PwMS. Sub-themes included adaptation; loss and grief; difficult emotions; reframing, re-evaluating and re-prioritizing; hope and optimism; empowerment and taking control; and self-awareness, greater understanding and personal growth.

**Conclusion:** Partners of PwMS who have undertaken lifestyle modification experienced a broad range of psychological adjustments. Whilst reflecting the potential difficulties that partners of PwMS may experience, this group experienced a range of positive psychological changes that add to the literature regarding partners' potential experiences and may provide hope for those in partnerships with people with MS. This study provides themes to potentially inform a quantitative study of a larger population of partners of PwMS.

## Introduction

Being in an intimate relationship with a person with multiple sclerosis (MS), a neurological condition with an unpredictable course, may have a substantial impact on the partner's quality of life. Partners of people newly diagnosed with MS in particular may not be in “hands-on” caring roles but may recognize a potential future change to their role, leading to a sense of uncertainty and a disruption to the identity they had taken for granted, from that of partner to that described as an anticipatory carer (Strickland et al., 2015). Partners of those with any chronic condition may experience a sense of worry about the person's well-being, their relationship and the future (Cheung and Hocking, 2004) and the partner's quality of life may be significantly affected (Figved et al., 2007; O'Connor and McCabe, 2011).

Existing research on partners of PwMS has largely focused on negative impacts of MS for the partner and has painted a rather bleak view of the future. From the time of diagnosis partners may experience levels of stress affecting their own physical and mental health and family function, similar to partners of those diagnosed with other life-threatening or disabling disease (Figved et al., 2007; Bogosian et al., 2009; Checton et al., 2012). Quality of life can be adversely affected for partners in a caring role (O'Connor and McCabe, 2011). Although PwMS may live healthy, active lives, 30% require a level of informal, unpaid caregiving at some point in their lives (Hillman, 2013). In up to 78% of cases this care is provided by a spouse or partner, compared with only 20% of care being provided by a partner for other disabled adults (Hillman, 2013). The daily requirements of caregiving can result in physical, psychological, emotional, social, and financial stressors (Buhse, 2008; Hillman, 2013). Those providing care may need to confront having to make significant changes to their working lives, may experience an impact on their personal and social lives (Bayen et al., 2015), and may have to adjust their living arrangements and other practical aspects of life (Strickland et al., 2015).

There is, however, an emerging body of literature regarding positive impacts of MS on partners, such as gaining insight into illness and hardship, personal growth, a re-evaluation of life's priorities and goals, and a greater appreciation of life and of one's own health (Pakenham, 2005). There may also be some degree of adversarial growth, that is, personal growth through adversity, where patients and their partners experience the trauma of having a chronic illness and subsequently find positive aspects together (Ackroyd et al., 2011). Partners and carers may also make their own personal health gains when, confronted with their own vulnerability to illness, consequently make lifestyle changes that protect their health. Carers may also make healthy lifestyle changes to ensure that they remain sufficiently healthy to care for the PwMS (Pakenham, 2005).

It is also possible that there are other potential positive experiences for partners. In recent years modifiable lifestyle factors have become increasingly recognized in the treatment of MS symptoms and disease progression (D'hooghe et al., 2010; Riccio and Rossano, 2015; Altowaijri et al., 2017; Cortese et al., 2017; Hempel et al., 2017). Our research group has investigated and published results from the Health Outcomes and Lifestyle In a Sample of people with MS (HOLISM) and Studying Outcomes of People attending MS retreats (STOP MS) studies. We have shown that participation in a residential lifestyle educational intervention (LEI) and/or engagement in lifestyle modification is associated with improved mental and physical health outcomes (Hadgkiss et al., 2013, 2015a,b; Jelinek et al., 2013; Marck et al., 2014; Taylor et al., 2014), improved quality of life and less disability in those affected by the disease (Jelinek et al., 2016a,b).

To date, previous partner research has sampled the population of partners of PwMS who have primarily adopted standard medical management only. For partners of people with MS who have undertaken lifestyle modification as an additional strategy to minimize disease progression, the impacts, both positive and negative, remain unexplored. This research is unique in that it focuses on the partners of this group of PwMS who have attempted to adopt major lifestyle interventions outside of the prevailing paradigm of MS management. Here we report aspects relating to the psychological shift experienced by partners.

### Aim

To explore and interpret the lived experiences of partners of PwMS who had attended a residential LEI, to understand partners' attitudes to and experiences of the effect of MS and any lifestyle interventions undertaken by the person with MS and themselves, on their life, relationship and view of the future.

## Methods

### Study design

Ours was a qualitative, interpretive, phenomenological study using semi-structured interviews. The philosophical foundation guiding the research was that of Interpretive Heideggerian Phenomenology (Converse, 2012) that assumed that the researchers' ideas and biases were acknowledged but not “bracketed” from the phenomenon that they were seeking to understand, that is, how participation in the LEI and involvement in lifestyle modification had affected the lives of participants.

### Participants

Eligible participants were: English-speaking, aged 18 years or more; had been in a spousal relationship for 12 months or more with a person with MS who was enrolled in the HOLISM study and who had attended a residential LEI in Australia, New Zealand, United Kingdom or Europe. Participants may or may not have attended the LEI with the person with MS.

### Sampling strategy

The existing HOLISM dataset consists of approximately 3,500 people of whom approximately 10% (350) had attended an LEI and of whom approximately 80% (280) indicated being partnered. The total population of those in the dataset who attended an LEI and indicated being partnered were assigned a number using a random number generator in Microsoft Excel and then sequentially invited to participate via email. Invitations were sent in groups of 10 and when interviews had been scheduled for those responding, another 10 invitations were sent. This was to ensure that those willing to participate could be interviewed without delay and at their convenience. We undertook some purposive sampling toward the end of recruitment in order to achieve representation by male and female partners.

The email to the PwMS contained information about the study and requested they forward the email, which contained an invitation for participation, to their partner. The invitation directed partners to a SurveyMonkey™ webpage containing participant information and an invitation acceptance. Partners were asked to reply whether they were willing to participate. If the answer was “no” they were directed to the last page of the survey which said “thank you for your time.” If the response was “yes,” the following pages contained a request for information to ensure eligibility and enable planning of interviews. Partners were asked to provide contact details, whether they had attended the LEI, the facilitator of the LEI and the name of the person with MS. Details of the person with MS were used to determine who had responded.

One hundred and three email invitations were sent. Twenty (19%) invitees declined participation. Seventeen (16%) declined by clicking on “I do not wish to participate” on the webpage. There was no ability to provide a reason for declining participation as the researchers felt it was imposition on those who did not wish to participate to request further engagement with the research project. Two (2%) replied by return email to the researchers and said they no longer had a partner and one (1%) said they had never been to a retreat. Fifty-nine (57%) email invitations to participate did not receive any response. Twenty-four (23%) partners indicated a willingness to participate but three (3%) partners were unable to be contacted again to arrange interview. Twenty-one (20%) interviews were conducted, six women and fifteen men. There were no repeat interviews. Durations ranged between 20 and 62 min with an average of 36 min. Ages of partners ranged from 28 to 79 years. The range of duration of spousal relationships was 5–51 years, and all relationships were heterosexual.

### Setting

Interviews were conducted between July and October 2016 via telephone or internet interface (Skype™). Interviewers were located in Melbourne, Australia. Interviewees were located in their home and were generally alone, although two participants indicated the person with MS could hear the discussion but played no role in the interview.

### Research team

Interviews were conducted by one of two female senior specialist medical practitioners (SN, KT) working in academic roles and with extensive experience in conducting interviews clinically and for the purpose of research. Both interviewers had individually, but not concurrently, delivered the LEI. Potential dual relationship with participants was avoided by ensuring the interviewer had not facilitated the LEI that the person with MS and, potentially, the partner had attended. Participants were aware that the interviewer was a researcher from the University of Melbourne who had been a facilitator of and had in depth knowledge of the content of the LEI.

### The interview

The interview was introduced with the following statement to give context to the participants. “The reason we are speaking with you today is that current research into partners of people with MS generally paints a fairly negative view of the experiences of people with MS and their partners. We are interested in speaking with partners of people who have been to a residential lifestyle workshop and modified their lifestyle to manage their MS to understand your particular experiences. Is there anything you would like to ask about the study and me before we commence?”

The interview consisted of three main questions:

1. How has MS and any lifestyle modification that you/your partner have undertaken affected your life?
2. How has MS and any lifestyle modification that you/your partner have undertaken affected your relationship with the person with MS?
3. How do you see your future and what has influenced this view?

### Data collection

All interviews were audio recorded digitally. Verbal consent to participate was obtained. Interviewers then followed a pre-designed interview schedule (Appendix A). Three broad questions were asked regarding the effect that MS and any lifestyle modifications had had on the partner's life, the effect on their relationship with the person with MS and the effect on their view of the future. Interview prompts were provided for the interviewer but participants were encouraged to expand as much as they desired.

Demographic data were collected directly from participants in the email response and during interview. To assess the level of disability of the person with MS, participants were asked “has the person with MS used a walking aid in the last 6 months?” This corresponds with Step 4 or above on the Patient Determined Disease Steps Scale, meaning that some form of mobility support is required at least some of the time (Hohol et al., 1995).

### Data storage and transcription

An independent company transcribed audio recordings of interviews in a de-identified manner. Participant information, digital recordings and de-identified interview transcripts were stored securely and password protected. Interview transcripts were not returned to participants but were read and edited by one of the interviewers (SN) while concurrently listening to the audio recordings to ensure accuracy of transcription.

### Data analysis

Data were imported into NVivo™ software. Each interview was descriptively coded followed by a search for themes and patterns across each interview. The first two authors (SN, KT) analyzed transcripts, coded the data and met regularly to discuss emerging themes derived from the data. Interviews were recoded and themes revised until researchers felt they adequately reflected participants' experiences. Coders determined when saturation of themes had occurred and sampling could cease. A third researcher, a practicing clinical psychologist (TW), not involved in the interview or initial coding phase, studied the data collected and confirmed themes. Some themes were broad constructs that emerged from many different kinds of expressions from participants. Other themes were more focused and related to very specific expressions from some participants. Due to this manner of theme development, it was not possible to quantify the proportion of participants expressing each theme. Quotations were reported that were thought to best represent the essence of each theme.

This paper analyses the major theme of psychological shift. The coding tree was:

Psychological shift

Adaptation
Loss and grief
Difficult emotions
Reframing, re-evaluating and re-prioritizing
Hope and optimism
Empowerment and taking control
Self-awareness/greater understanding/personal growth.

### Rigor

Trustworthiness and auditability were maximized by the use of an independent third party for transcribing audio recordings verbatim and researchers ensured accuracy of transcriptions by simultaneously listening to recordings while reading transcripts. Accuracy was enhanced by verbatim textual quotes used as exemplars of the analytic outcome in the presentation of findings. The authors kept a systematic audit trail comprising interview notes made following interviews, sequential versions of software demonstrating evolution of themes, notes of researchers' discussions of themes and personal reflections of the data analysis. The researchers involved in interviewing and analysis discussed their preconceived beliefs about PwMS, partners of PwMS, and the LEI, in order to be consciously aware of their position within the research itself. No repeat interviews were conducted.

### Ethics

Ethics approval was obtained from the University of Melbourne Human Research Ethics Committee ID number 1545280.1.

## Results

Demographic and MS related data are shown in Table 1. At times partners referred to the Overcoming Multiple Sclerosis (OMS) programme of lifestyle modification to which their partner/they subscribe and to the residential LEI as “the retreat.” The assigned interview number (P) and gender (M = male, F = female) of participants are indicated following each quotation.

**Table 1:** Participant information.

**Gender (PwMS)**	**Age (P)**	**Years since LEI (PwMS)**	**LEI attendance (P)**	**Region**	**Employment (P)**	**Type of MS (PwMS)**	**Years of relationship**	**Years of MS (PwMS)**	**Disability (PwMS)**	**Walking aid (PwMS)**
Male	30-39	>5	Yes	Australia	Full time	RRMS	11–20	5–10	No	NA
Female	20–29	2–5	Yes	UK	Full time	PPMS	1–10	5–10	No	NA
Male	40–49	1–2	No	UK	Full time	Unsure	21–30	0–5	No	NA
Male	50–59	1–2	Yes	UK	Part time	RRMS	21–30	0–5	No	NA
Female	40–49	>5	Yes	Australia	Part time	RRMS	1–10	11–20	No	NA
Male	70–79	>5	Yes	NZ	Retired	SPMS	>50	11–20	Yes	Wheelchair
Male	60–69	>5	No	Australia	Retired	SPMS	41–50	11–20	Yes	Stick
Male	40–49	2–5	Yes	Australia	Full time	Unsure	1–10	5–10	No	NA
Male	20–29	2–5	Yes	Australia	Part time	SPMS	1–10	0–5	No	NA
Male	60–69	>5	No	NZ	Full time	Unsure	21–30	5–10	No	NA
Female	60–69	>5	Yes	NZ	Full time	PPMS	41–50	11–20	Yes	Stick
Male	60–69	2–5	Yes	UK	Retired	CIS	31–40	0–5	No	NA
Female	40–49	>5	No	NZ	Full time	RRMS	1–10	5–10	No	NA
Male	30–39	1–2	No	Australia	Full time	Unsure	1–10	5–10	No	NA
Male	60–69	>5	No	Australia	Retired	Unsure	41–50	11–20	No	NA
Male	50–59	1–2	No	NZ	Unable	Unsure	31–40	0–5	No	NA
Female	20–29	1–2	No	UK	Maternity	RRMS	1–10	0–5	No	NA
Female	50–59	2–5	No	UK	Full time	PPMS	11–20	5–10	Yes	Stick
Male	60–69	2–5	Yes	NZ	Retired	RRMS	41–50	> 40	No	NA
Male	60–69	2–5	No	Europe	Full time	RRMS	11–20	5–10	Yes	Stick
Male	70–79	>5 years	Yes	Australia	Retired	Unsure	21–30	11–20	Yes	Other

*MS, multiple sclerosis; P, partner; PwMS, person with multiple sclerosis; NZ, New Zealand; NA, not applicable; RRMS, relapsing remitting MS; PPMS, primary progressive MS; SPMS, secondary progressive MS; CIS, clinically isolated syndrome*.

*Disability question: “Has the person with MS used a walking aid in the last 1 week?” Type of MS was as described by the partner from recollection*.

### Adaptation

Many partners reported that a major psychological shift that they strived for or had achieved was the incorporation of MS into the life of the partnership. For some, at times, MS was seen as only one of many of life's challenges.

*My view of the world is that you play the cards you're dealt. We were dealt quite a few good cards, but we were dealt one bum card that was MS. So hey, that's part of the cards. You just live with it…and…we are two people with a whole load of characteristics and we've got a relationship that's built in the light of those. No, it's just part of our life (M,P14)*.

For some, MS had imposed constraints on their lives but they had come to accept and incorporate these changes into their lives.

*MS is definitely part of our reality and it's working out how you kind of work with the limitations, but we live a full life within those limitations (F,P10)*.

At times MS had imposed some limitations and constraints on the couple's lives and they had adapted to these changes, incorporated them into their perspective, and modified their plans accordingly. These modifications were not seen as burdensome.

*We're happily married and we want to live life and go and do great things. Working towards retirement, getting the kids out of the house, seeing some more of the world and relaxing and having a great time. My partner will go and do physio and, maybe she won't be trail running ultra-marathons with me through the bush in Australia, but fingers crossed she gets better and gets some more mobility and will go and do great stuff. If she doesn't, we'll find another way of doing it (M,P21)*.

*We want to travel. What we've done is slightly modify our thoughts on how we travel because, physically, it's not so easy…so the details are different but our vision of the future is still the same (M,P14)*.

### Loss and grief

Some participants described making changes in their lives and adapting to illness but expressed that this had been very difficult and that they had experienced a sense of loss and associated grief. Some experienced the loss of what they had previously done as a couple before MS.

*I think the regrets are probably around the things that we used to do. We used to sail and tramp [bushwalk] and just do lots of physical stuff. I think that that's had to be modified and it's very different (F,P3)*.

*Walking is a big problem. We still go to France every year, but we used to love walking through the mountains, and that can't be done anymore. So that's a huge change. We used to horse ride, that can't be done anymore. We used to own a farm - so we had to sell the farm…Things like that, which have been huge changes (M,P18)*.

Others described a sense of loss of control over their lives as a couple, not knowing what was to happen next.

*Well low control. Let's not exaggerate, very low control. There's not a lot I can do really…I'll just roll with it but I don't feel I've got any control over (the future) now whereas before maybe I had some (M,P13)*.

For some the experience of loss extended to a loss of their sense of self due to the increased responsibilities of caring for the person with MS and their family.

*Because we have children I can't just disappear off and do my own thing…I feel like I have probably stepped back from my own life slightly. I don't have a massive problem with it but I am aware that I've done that and he's aware he's done that too, it's just a difficult situation at the moment with a young family (F,P20)*.

For some, there was a sense of sacrifice and loss of what the future held for them personally.

*But the impact for me has been around I don't get to see all of the things I'd like to see. I don't get to stay in places as long as I'd like because she runs out of puff and says look…we've got to go and you go okay (M,P13)*.

*I've decided that this is how it will be until our children are older that I have just given up a little bit of my life in order for him to focus on what he needs to do to try and get better and stay healthy (F,P20)*.

And at times, for some partners there was a sense of sacrificing their own well-being.

*I think basically he realized at the same time that I realized that since he had been diagnosed with MS I had literally done everything and had stopped looking after myself and had just focused on him being well (F,P20)*.

### Difficult emotions

While loss and grief were apparent for some, others reported dealing with other difficult emotions. For partners of PwMS with significant disability, many challenges were described. Some of the challenges had significant impact on the partner's psychological state.

*Well, I mean she's got it but it's colonised me as well. That's the word I would use. It's colonised me in a whole lot of secondary ways (M,P13)*.

Apprehension and frustration due to the physical and emotional challenges the disease presented were experienced.

*So suddenly you'll get a shift and an explosion in the kitchen or something when she drops something and that I find very disconcerting…You're thinking hey the clock's ticking, the bomb's going to go off any minute even when it doesn't. So - it's made me apprehensive…yeah, occasionally we snap at each other. I sometimes have a dig at her and that's not a thing I'm proud of…if I'm going to have an excuse for it or an explanation, it's the frustration with the disease (M,P13)*.

Partners experienced uncertainty and guilt around trying to determine the best way to assist when the person with MS was struggling physically or emotionally.

*I'm not a very expressive person, so she found it difficult. So that's been an issue, like she might say you didn't even ask how my day was today. So, I believe that is a repercussion of the disease…so, there's some additional strain in that regard (M,P19)*.

*I feel guilty some of the time because part of it is I don't want her to not do stuff because I know that if she stops doing stuff then you lose the capacity. If you don't use you lose it sort of thing…sometimes I know she's really struggling…but I think well it's better for her (M,P13)*.

*That will mainly be because I'm not so in touch with my own emotions, but then I find that my partner's so in touch with her emotions. Sometimes I guess I'm a good listener and then when my partner wants a response, sometimes I just really don't know what to say (M,P4)*.

Partners described experiencing the pain of witnessing the physical deterioration and the frustration and limitations the person with MS experienced.

*She was very dynamic, she had her own career, she worked very hard, and she travelled… some days it's a very frustrating situation for her. I've seen her reduced to tears and…of course that impacts on me…I wish it would go away. I just hate it (M,P13)*.

*There are other people with his type of MS who would have already gone to more assistance than just the stick, but he's so determined, and like I say, very, very stubborn, so he basically said he's going to keep going until he just absolutely cannot move anymore under his own steam. So that's just kind of hard to sit and watch…so that for me is the hardest thing (F,P17)*.

### Reframing, re-evaluating and re-prioritizing

However, despite these difficult emotions and experiences, there were often positive changes described by many partners, with MS representing an opportunity for seeing life in a different way, re-evaluating and focussing on what was important in life and their new priorities. Some had used the lifestyle modification programme to shift their focus away from the diagnosis and the illness to what could be done, and had reframed their circumstances as an opportunity for improving health.

*The main thing is that our focus is not on the MS. We never ever really talk about it because it's just part of our lives now. We instead focus on the OMS side of things, so diet, meditation, focus on those rather than the actual MS diagnosis (F,P7)*.

Many partners described that they had used MS, and the uncertainty that the illness had caused in their lives, as an opportunity to re-evaluate what was important in life and to get their life's priorities in order.

*I think my day-to-day outlook is different…my work is secondary to family and things like that. So, it's certainly contributed to the way I spend my time. I'm shifting focus less on work (M,P19)*.

*Like I say this whole working for money and new cars and great houses, what difference does it make? Because you don't know what's going to happen and take advantage of time with each other a little bit more and actually appreciate what you have with each other a lot more. That…was a little bit of an awakening (F,P17)*.

*You do evaluate what you want to do and what's important in your life and what do you want to carry on doing and what you don't want to do (M,P8)*.

Some even felt that their relationship wouldn't have eventuated if not for the re-evaluation that occurred during the MS journey.

*We weren't together at the start so we don't know life without it. I think it's interesting. He was separated about a year after his diagnosis. He often says it was the best thing that ever happened to him, getting MS, because now he realises what's important and what's not (F,P1)*.

Following the re-evaluation and re-prioritization, some partners had used the experience of living with MS and the programme of lifestyle modification as an opportunity for significant positive psychological shift.

*We both made a decision to use the MS, not as a negative like it always had been, but start looking at it as a positive and make a different lifestyle change completely…the positive thing is then seeing that because of the MS we've been able to change our lives in a very positive way as far as health and wellbeing overall, the diet, the meditation…and exercise. Just the whole lifestyle, stepping back and actually taking stock of what is important (F,P17)*.

### Hope and optimism

Partners described the benefit of the lifestyle modification and the residential LEI (retreat) in providing hope for the person with MS, and the resultant beneficial effect of this new-found hope in their own lives.

*It appears to us that he's getting better every year. So we look back and go wow, can you believe it? You wouldn't have thought 10 years on that you'd be healthier than you were many years ago. So that makes it easy…the big thing I always say…is there's no hope initially and what the programme gave him, was that exactly. The retreat was just the turning point in his life…we came away with hope (F,P1)*.

*I guess my thought of our future has never changed really from before she was diagnosed and after she was diagnosed. If anything, we do more now than we did before. I see it probably more positive that it was before (M,P4)*.

*Sometimes he'd be really positive, sometimes he'd be quite down about it. Then when he went on the retreat and when he came back from the retreat he was just like a completely transformed person (F,P20)*.

### Empowerment and taking control

For some, the knowledge and experience of the lifestyle modification programme led to the partner and the couple experiencing a new sense of empowerment to deal with the disease.

*We feel very empowered and just really lucky that we were able to have the know how to find out about this. Because there's a lot of people who go to the doctor and they are told by the doctor you have MS, and that's all you're told and you're told you need to take this medication and see what happens (F,P20)*.

*We're very rational in our thinking, scientifically minded having the evidence base and so on, and certainly the OMS regime follows that. It isn't quackery, if you like. It's evidence-based (M,P5)*.

*We actually feel that MS doesn't run us. We run the MS (M,P14)*.

Partners felt empowered to manage their own health as well.

*I almost in a way feel glad that it happened which I would never have thought I would ever say that…I feel quite empowered…to have the knowledge and the understanding that I can treat myself in a way that will prevent or will increase my chances of preventing a huge host of conditions, illnesses, mental health (F,P20)*.

The empowerment provided by the knowledge led to a sense of control over the disease and the future for many.

*We have a feeling of having taken control, that MS doesn't control us. Also I think that the huge change that's occurring in everyone's attitude to health - taking much more personal engagement with it (M,P14)*.

*Yeah I actually feel more in control with making this decision not to work for someone else and take charge of our lives (F,P17)*.

### Self-awareness/greater understanding/personal growth

Some partners expressed that the opportunity to re-evaluate and reprioritize and to adopt the lifestyle modification programme had led to contemplation of other aspects of life, their relationship and their health.

*The 21st century is the century of the system; I'm fascinated by that. It is things like the amazing interactions between the health of the gut fauna, the biome, and mental health; so suddenly looking at the whole person, which OMS does, fits with a very enlightened understanding of medicine. I found that one of the most reassuring features about it. That it's rooted in actually looking at, first of all, the person rather than the disease, and then the disease as a consequence of many things, that's influenced by many things (M,P14)*.

*But I think the more you do all of those things, the more you actually understand the mind body connection and stuff, the more you take responsibility for your condition and work towards [getting] better and I'm a firm believer in that. So I think had he not done it (the retreat), I think the whole thing about trying to find a cure from other people…would have been very much part of his mental state, whereas now he understands that he's at the same time pretty much part of the equation (F,P10)*.

*I think (the retreat) helped him see the world differently…I think he was less judgmental of people and…a really useful thing…it really helped our relationship. So I don't think he would be the person he is today if he didn't have MS (F,P10)*.

*At the retreat…I was asked to tell my partner how I felt about him…some quite personal questions that we had to sit and discuss, and we had never discussed things particularly at that level and to that depth (F,P3)*.

*Because of MS actually and the diagnosis, we communicate a lot better now as well. We're very open with each other, because [partner] was quite a closed book but now he's much more open about how he's feeling, how his body is feeling, and we talk about it. Every decision is our decision and even when I talk about MS I always say we, I never say [partner]. It's always been a thing; I always refer to everything we do as we (F,P7)*.

## Discussion

The negative psychological impact on partners of people with MS has previously been well described in the literature and may be similar to the experiences of partners of those with other degenerative neurological conditions. The effects of the uncertainty of disease progression and the potential of the disease to cause significant disability, is well documented in the literature. For conditions such as motor neuron disease, this uncertainty has been described as throwing the “family into total uncertainty, since it is not possible to predict in advance what will happen in the course of the disease” (Cipolletta and Amicucci, 2015).

Our study found that partners described a variety of difficult emotions and painful experiences and they expressed the negative impact that these experiences had on their psychological state. Some partners expressed anxiety and frustration. Uncertainty and worry about the disease and what the future holds are commonly reported themes amongst partners of those with MS even in the early stages of the illness (Bogosian et al., 2009; Boland et al., 2012) and existential uncertainty is known to be a strong contributor to psychological distress for partners of those with MS and other diseases (Gullick et al., 2017). Unclear boundaries and unknown rules of how to assist have been reported for those in relationships with new conditions and new constraints on physical activities (Gullick et al., 2017). In our study, some partners described apprehension, uncertainty and guilt around not knowing how to help their loved one; those observing deteriorating function were sometimes torn between assisting and allowing the person to persevere to maintain independence. Uncertainty at a psychological level is known to result in a negative effect on adjustment (Giovannetti et al., 2017).

A sense of loss may manifest in various forms, such as the loss of perceived certainty about the future, an inability to plan, and a sense of helplessness (Bogosian et al., 2009). In our study some partners expressed feelings of loss regarding the things the couple used to do together that they could no longer do, such as travel and physical activities; what they themselves would not be able to experience in the future due to the constraints that the illness imposed; the loss of ability to concentrate on their own health and life goals; and the loss of freedom associated with the increased demands of their role.

Some partners expressed distress, sadness and pain when witnessing their partner's deterioration and subsequent loss of independence. Previous studies have also found that partners in a caregiving role experienced high levels of distress and reduced quality of life (Figved et al., 2007), especially partners who had experienced a longer duration of caregiving and those who cared for a person with MS with moderate or severe symptoms and an unstable disease course (Aronson, 1997). Although many aspects of carer burden across all illnesses have been described in the literature, some of the losses may be more pronounced for partners of those with MS where physical disability may occur at quite a young age and the partner subjugates their own needs, at least temporarily, to accommodate the person with MS. In our study there were some clear expressions of the sadness and difficulties around these losses.

Adapting to illness, for both the person with the illness and their partner, is well explored in the literature. Various psychological strategies that contribute to emotional and cognitive adaptation to MS include humor, optimism, flexibility, acceptance of a “new normal,” perseverance and self-compassion (Silverman et al., 2017). In line with this previous literature, many of our participants expressed acceptance, had incorporated MS into their daily lives, seeing it as only one of life's challenges. Many demonstrated strategies associated with adaptation to illness such as a positive outlook, optimism and flexibility, knowing that they could adapt to whatever challenges may come their way. Also previously described are the somewhat positive aspects of MS for partners in a caregiving role, such as a sense of finding benefit despite fairly overwhelmingly negative circumstances, such as “the realization that life could be worse” and “I don't worry about material things too much” (Pakenham, 2005).

Adaptations described by some participants in our study differed significantly from these previously described adaptations in that they experienced psychological benefit beyond this adversity-related benefit-finding, describing MS much more positively as an opportunity to re-evaluate priorities in life and they experienced genuine positive psychological change. These positive psychological changes translated into real life changes. Rather than the limitations of working opportunities that are frequently experienced by those in a caregiver role (Hakim et al., 2000; Bogosian et al., 2009; McKenzie et al., 2015), some partners had taken the opportunity to resign from stressful jobs, shift their location of residence to enhance their lives, adopt lifestyles that were mutually beneficial to both the person with MS and the partner and genuinely improve their own health, seemingly shifting from finding benefit through adversity to finding benefit from a new and challenging life that they had actively chosen.

Participants in this study were partners of PwMS who had undertaken a residential LEI (retreat) who had significantly modified their lifestyles and many of the partners had also adopted the lifestyle interventions. Partners observed improvements in the health of the person with MS and their own health that had added a further dimension to these partners' experiences of MS and had led them to have hope that good health may continue into the future. Previous literature has described low rates of adherence to self-management regimens and that self-management may often be seen as a burden to those with chronic illness and may add to psychological distress (de Ridder et al., 2008). Partners in our study frequently expressed that the self-management programme gave them genuine hope of improved health and hope regarding the future. Genuine hope has not previously been described for partners of PwMS.

The hope experienced by some partners in our study, at times, extended to confidence that their futures were not unpredictable and there was no reason to doubt that observed benefits could continue into the future. Rather than the loss of control so frequently expressed by partners of people with unpredictable illness (Boland et al., 2012), some partners experienced a greater sense of control. This sense of control was assisted by the evidence-based information they had received, the observed health benefits both for the person with MS and themselves, and the belief that these benefits would continue.

For some, this improved sense of confidence in the future developed into a sense of having regained control of their lives and having taken control of the illness rather than the illness having control of the person with MS and them. They experienced a sense of empowerment that they could influence their futures and that the control of the illness was back in the couple's hands.

For some partners in our study their experiences with MS and the lifestyle modification programme had given them the opportunity to reflect on deeper issues and to further understand the mind body connection. Some experienced new found control over their own health and were advocates of the shift of responsibility of health back to the individual. Some experienced greater self-awareness, witnessed personal growth in the person with MS and experienced their own personal growth and growth within the relationship.

## Limitations

Interviewees were all English speaking so there were no experiences reported from those of non-English speaking partners. The non-participation of people with MS who did not respond or declined participation may have introduced responder bias. Partners willing to participate in the study may have self-selected and had more positive experiences to report than those who did not participate. The researchers' in-depth understanding of the LEI may have affected access to the “field” of potential participants as respondents may have been more willing to share their experiences with these researchers whom they perceived as sympathetic to their situation and may, in turn have affected the information that participants were willing to share.

The study included only current partners of PwMS, therefore the experiences of partners whose relationships had ended were unable to be explored. No same sex partners participated and the researchers were unable to undertake purposive sampling to account for relationship type, as the data contained no reference to the gender of the partner. Partners were asked what type of MS the person with MS had been diagnosed with. These data were based on recollection and not medically confirmed, but were felt to not significantly affect results. In two cases the person with MS was present during the interview. The presence of the person with MS may have affected the responses given to some questions.

## Conclusion

While some partners expressed somewhat predictable psychological shifts previously described in the literature, such as difficult emotions and a sense of loss and sacrifice, many partners experienced acceptance and adaptation and some partners expressed a body of new and encouraging psychological and philosophical changes. Whilst no causal connection is drawn between the lifestyle intervention, the degree to which lifestyle modification has been adopted or the severity of MS and the themes that emerged, the experiences reported by this unique group of partners occurred in the context of undertaking lifestyle-modification and included hope, optimism, a sense of empowerment and taking control, confidence and personal growth. These contemporary qualitative findings should inform clinicians who may consider the benefits to both PwMS and their partners of engagement with lifestyle modification and emphasize the sense of control, empowerment, hope and confidence that may follow. This study also provides themes to potentially inform a quantitative survey study of a larger sample of partners of PwMS.

## Ethics statement

This study was carried out in accordance with the recommendations of the University of Melbourne Human Research Ethics Committee. As participants were never met in person, and telephone interviews were conducted and recorded, all subjects were provided with written participant information via email and gave verbal recorded informed consent during the interview.

## Author contributions

Accountability, manuscript revision and approval, and concept development: SN, KT, TW, GJ, CB, and AD. Manuscript drafting, data acquisition and analysis: SN, KT and TW.

### Conflict of interest statement

GJ receives royalties for the book Overcoming Multiple Sclerosis which outlines the pillars of the lifestyle educational intervention. KT, SN, and GJ have received remuneration for facilitating the lifestyle educational intervention. The other authors declare that the research was conducted in the absence of any commercial or financial relationships that could be construed as a potential conflict of interest.
